# A study of road rage in India

**DOI:** 10.1192/j.eurpsy.2021.1884

**Published:** 2021-08-13

**Authors:** P. Jain, V. Mudgal, V. Niranjan, V. Pal

**Affiliations:** Psychiatry, MGM Medical College, INDORE, India

**Keywords:** road rage, Driver anger, Indian Drivers, Aggression

## Abstract

**Introduction:**

Road rage is a term used to describe driving usually extreme in nature. There seems to be a multifactorial relationship between the situational characteristics of an anger provoking road situation and the feelings of anger and road behaviour.

**Objectives:**

To examine driver anger with regards to various sociodemographic parameter.

**Methods:**

282 participants completed an internet-based survey including sociodemographic profile, anger assessment while driving using the Deffenbacher Driver Anger Scale, details of the driving. Participants were recruited through networks of authors, institution. The survey was disseminated through social media applications and email by snowball sampling method.

**Results:**

Mean age of the sample was 26.1 years with age group 24-29 years making half of the population. Majority sample were males (62.1%), graduates (53.2%), professionals (45.7%), urban locality based, nuclear family type. People experienced greater anger on Defenbacher likert scale for the following situations, when Someone is driving very close to your rear bumper (mean= 3.09), Someone cuts in right in front of you on the motorway(mean= 3.44), Someone cuts in and takes the parking spot(mean= 3.19), Someone coming towards you does not dim headlights at night(mean= 3.26), driving behind a vehicle smoking badly or giving off fumes(mean= 3.38).

**Conclusions:**

The results revealed a prevalence of high anger scores amongst Indian drivers. The rage didn’t vary significantly within gender, locality, type of vehicle, however the anger scores were significantly higher in younger population. Strategies targeting at driving safety and reducing road rage should be implemented by authorities with sensitization of the drivers.

**Disclosure:**

No significant relationships.

